# Mesenchymal Stem Cells Extract (MSCsE)-Based Therapy Alleviates Xerostomia and Keratoconjunctivitis Sicca in Sjogren’s Syndrome-Like Disease

**DOI:** 10.3390/ijms20194750

**Published:** 2019-09-25

**Authors:** Ghada Abughanam, Osama A. Elkashty, Younan Liu, Mohammed O. Bakkar, Simon D. Tran

**Affiliations:** McGill Craniofacial Tissue Engineering and Stem Cells Laboratory, Faculty of Dentistry, McGill University, Montreal, QC H3A 0C7, Canada; ghada.abuelghanam@mail.mcgill.ca (G.A.); osama.elkashty@mail.mcgill.ca (O.A.E.); Younan.liu@mcgill.ca (Y.L.); mob11@case.edu (M.O.B.)

**Keywords:** Sjogren’s syndrome (ss), autoimmune diseases, biologic therapy, bone marrow, cell extract, lacrimal gland, mesenchymal stem cells (MSCs), non-obese diabetic mice (NOD), salivary glands, submandibular glands

## Abstract

Sjogren’s syndrome (SS) is an autoimmune disease that manifests primarily in salivary and lacrimal glands leading to dry mouth and eyes. Unfortunately, there is no cure for SS due to its complex etiopathogenesis. Mesenchymal stem cells (MSCs) were successfully tested for SS, but some risks and limitations remained for their clinical use. This study combined cell- and biologic-based therapies by utilizing the MSCs extract (MSCsE) to treat SS-like disease in NOD mice. We found that MSCsE and MSCs therapies were successful and comparable in preserving salivary and lacrimal glands function in NOD mice when compared to control group. Cells positive for AQP5, AQP4, α-SMA, CK5, and c-Kit were preserved. Gene expression of AQP5, EGF, FGF2, BMP7, LYZ1 and IL-10 were upregulated, and downregulated for TNF-α, TGF-β1, MMP2, CASP3, and IL-1β. The proliferation rate of the glands and serum levels of EGF were also higher. Cornea integrity and epithelial thickness were maintained due to tear flow rate preservation. Peripheral tolerance was re-established, as indicated by lower lymphocytic infiltration and anti-SS-A antibodies, less BAFF secretion, higher serum IL-10 levels and FoxP3^+^ T_reg_ cells, and selective inhibition of B220^+^ B cells. These promising results opened new venues for a safer and more convenient combined biologic- and cell-based therapy.

## 1. Introduction

Sjogren’s syndrome (SS) is a common progressive autoimmune disease that affects females predominantly [1,2,3]. The prevalence of SS is variable worldwide; ranging from 0.1% to 0.72% of the population [4,5,6,7,8,9,10,11]. SS progresses slowly and patients exhibit clinical symptoms years after the disease onset [12]. The immune system targets epithelial tissues, infiltrates it with lymphocytes, and later forms autoantibodies against glands antigens [3,13,14,15,16]. The aberrant immune dysregulation leads to the destruction of epithelial tissues, especially salivary and lacrimal glands, and to several extra-glandular manifestations. The secretory function of the glands diminishes gradually resulting in dryness of the mouth (xerostomia), eyes (keratoconjunctivitis sicca), and organs containing exocrine glands, such as the nose and vagina [17,18,19,20]. The current SS management is symptomatic-based to alleviate the dryness severity and complications [21,22]. However, patients with systemic involvement and serious complications are prescribed immunosuppressant and disease-modifying antirheumatic drugs [23,24,25]. Unfortunately, the current management is not adequate nor satisfactory, leading to a compromised quality of life [26,27,28].

MSCs are multipotent cells that can self-renew and give rise to specialized cell types such as bone, cartilage, and muscles [29,30,31,32]. They were firstly isolated from the bone marrow, and later were extracted from various tissues, including peripheral blood, umbilical cord, adipose tissue, periodontal ligaments, and dental pulp [30,33,34,35]. Under normal homeostasis, MSCs are actively involved in the connective tissue maintenance. During tissue repair, they are responsible for secreting bioactive molecules that result in tissue regeneration and restoration [36]. MSCs are hypoimmunogenic because they lack the expression of MHC II and they express low levels of MHC I [37,38]. MSCs have demonstrated promising therapeutic potentials when used in different diseases and in tissue regeneration. They were successfully deployed in the management of neural injuries, GVHD (graft versus host disease), cardiac regeneration, and most importantly autoimmune diseases [39,40,41,42,43,44,45,46,47,48,49,50,51,52,53,54,55,56,57,58]. MSCs display a unique combination of immunoregulatory/immunosuppression, tissue regeneration/repair, and anti-fibrotic properties [59,60] which make them a suitable therapeutic modality for autoimmune diseases. 

MSCs are well documented for their immunomodularity and anti-inflammatory properties [61,62,63,64]. Several studies have reported that MSCs suppressed T and B cells proliferation when injected at the peak or at the onset of the disease [65,66,67]. However, Xu et al. have reported a defective MSCs immunoregulatory function in SS patients and NOD mice [50]. They incubated BM-derived MSCs from NOD mice and SS patients with PBMCs (Peripheral Blood Mononuclear Cells), a significantly higher proliferation rate of PBMCs was found in comparison to MSCs isolated from healthy donors. Thus, the previous findings rationalize the replacement of MSCs in NOD mice or SS patients with adequately functioning ones from healthy donors to compensate for the defective immunoregulation function. Nonetheless, the utilization of MSCs in treatments is not risk-free. These cells possess an attractive self-renewal and unlimited proliferation capacities, but these characteristics can be unpredictable and uncontrollable in vivo. MSCs might form tumors or enhance the progression of an existing one [68,69]. Therefore, transforming MSCs into extract/lysate can eliminate, theoretically, the tumorigenic risk. Our group and others have reported the therapeutic potentials of bone marrow cell extract (soup) in the management of irradiation-induced and SS damage of salivary glands and myocardial infraction, respectively, indicating the success of the concept [70,71,72]. Yet, to the best of our knowledge, the efficacy of MSCs extract (MSCsE) has not been tested in any field. 

The main aim of this study is to evaluate the efficacy of MSCsE in preserving the exocrine function of the salivary and lacrimal glands in NOD mice in comparison to MSCs. We hypothesized that MSCsE treatment executes this task via two mechanisms. Firstly, through their trophic and regenerative capacities, and secondly, by re-establishing peripheral tolerance which protects the glands against the autoimmune attack and eventually preserving the tissues from the autoimmune destruction. 

## 2. Results

### 2.1. MSCs and MSCsE both Preserved Salivary and Lacrimal Gland Functions, Preserved Specialized Cells, and Upregulated Key Genes in the Gland Restoration

**Figure 1:**
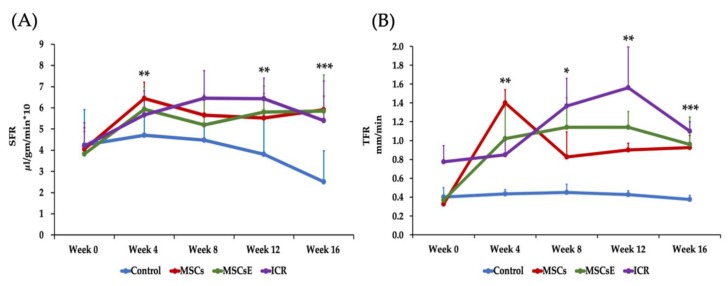
Salivary and lacrimal glands function represented as SFR (Salivary Flow Rate) and TFR (Tear Flow Rate), respectively. SFR and TFR were assessed pre-treatment at week 0 (8-week-old) then 4, 8, 12, and 16 weeks post-treatment. (**A**) SFR was determined by volume of saliva/min/gm body weight (multiplied by 10 for simplicity). Control group showed a continuous decrease of SFR (lost almost 47% of SFR at week 16 in comparison to the highest reached level, week 4) whereas MSCs-/MSCsE-treated groups maintained a significantly higher SFR (maintained almost 75–100% of SFR at week 4) than the control, their results were comparable to each other, and to the ICR group, (*n* = 5–12). (**B**) TFR was determined by length of wetted phenol red thread in mm/min. Control group showed a continuous decrease of TFR; whereas, MSCs-/MSCsE-treated groups maintained significantly higher TFRs that are comparable to each other and to the wild type ICR group. *p ≤ 0.05; **p ≤ 0.01; ***p ≤ 0.001, *n* = 3–6. All data were presented as mean ± S.D. Control: saline-treated; MSCs: Mesenchymal stem cells; MSCsE: Mesenchymal stem cells extract.

SFR (Salivary Flow Rate) and TFR (Tear Flow Rate) are objective measurements of the glandular function and are important tools for the evaluation of the treatment success. SFR and TFR were measured at five consecutive time points: pre-treatment at week 0 (8-week-old) then 4, 8, 12, and 16 weeks post-treatment. Upon analysis of the SFR, the untreated control NOD mice showed a steady deterioration in SFR that reached its lowest level at week 16 (24-week-old). The MSCs-/MSCsE-treated groups showed higher SFRs than the control group at all time points and comparable to that of the ICR group. Statistical analysis revealed that SFR levels were significantly higher at 4, 12, and 16 weeks post-treatment with 75–100% preservation of the function in comparison to the highest level recorded at 4 weeks post-treatment (Figure 1A). Both treated groups showed a significant increase in TFR (which represents the lacrimal gland function) at week 4 post-treatment. Afterwards, TFR declined significantly for MSCs but was maintained for the MSCsE. However, TFR for both treatments was significantly higher than the control group and comparable to ICR group especially the MSCsE-treated group (Figure 1B). 

**Figure 2:**
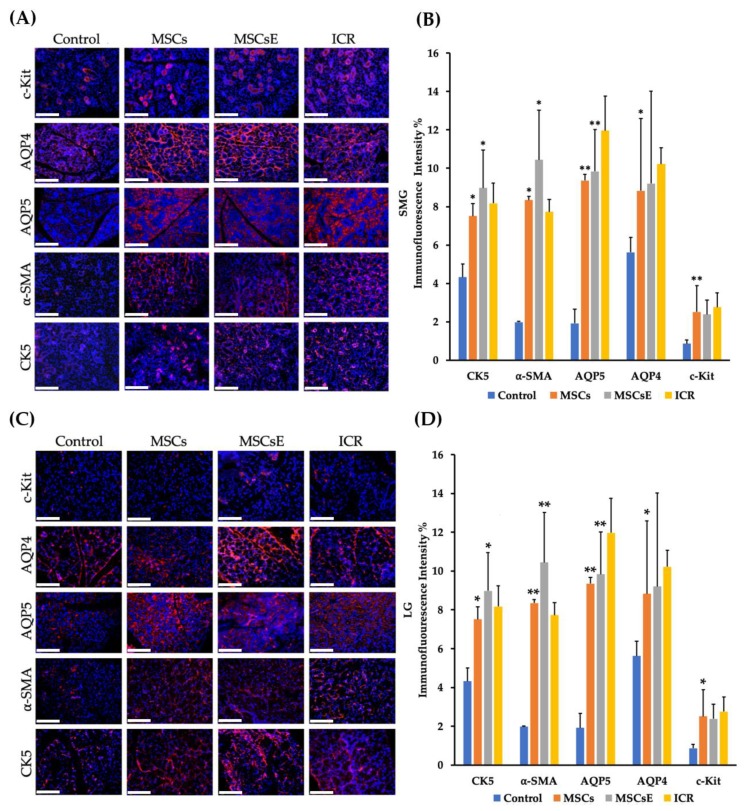
Special cell subpopulations in submandibular (SMG) and lacrimal glands (LG) were evaluated by immunofluorescence staining at 16 weeks post-treatment. (**A**,**C**) SMG/LG immunofluorescence staining, respectively, positive for AQP5 (marker for water channel protein expressed by acinar cells in SMG and acinar/ductal cells in LG), α-SMA (marker for myoepithelial cells), AQP4 (marker for acinar and ductal cells), CK5 (marker for ductal/progenitor cells), and c-Kit (marker for stem/progenitor cells) were tested in frozen sections, scale bar = 148 μm. **(B**,**D**) Quantification of protein immunofluorescence expression levels in submandibular/lacrimal glands, respectively, from 4–6 random fields/glands by Image J software. MSCs-/MSCsE-treated groups showed higher intensities for all the tested markers when compared with the control group. All images were randomly taken at 200× magnification. *p ≤ 0.05; **p ≤ 0.01, *n* = 3–6. All data were presented as mean ± S.D. Control: saline-treated; MSCs: Mesenchymal stem cells; MSCsE: Mesenchymal stem cells extract.

To further explain this preservation of function, we assessed multiple markers, genes, and factors involved in the saliva/tear formation, secretion, and glandular regeneration. Immunofluorescence staining was used to evaluate the expression of special cell subpopulations in both submandibular and lacrimal glands. We found significantly higher cells positive for AQP5 (Aquaporin 5, a marker for water channel protein to identify acinar cells in submandibular glands and acinar/ductal cells in lacrimal glands), AQP4 (Aquaporin 4, a marker for water channel in acinar and ductal cells for both glands), α-SMA (alpha Smooth Muscle Actin, a marker for myoepithelial cells), CK5 (Cytokeratin 5, a marker for ductal/progenitor cells), and c-Kit (a marker for stem/progenitor cells) in the MSCs-/MSCsE-treated groups than the control group (Figure 2A–D).

### 2.2. MSCs/MSCsE Treatments Promoted Proliferation, Elevated Systemic EGF Levels, and Modified Specific Key Genes in Glands Function, Proliferation, Regeneration, and Apoptosis

**Figure 3:**
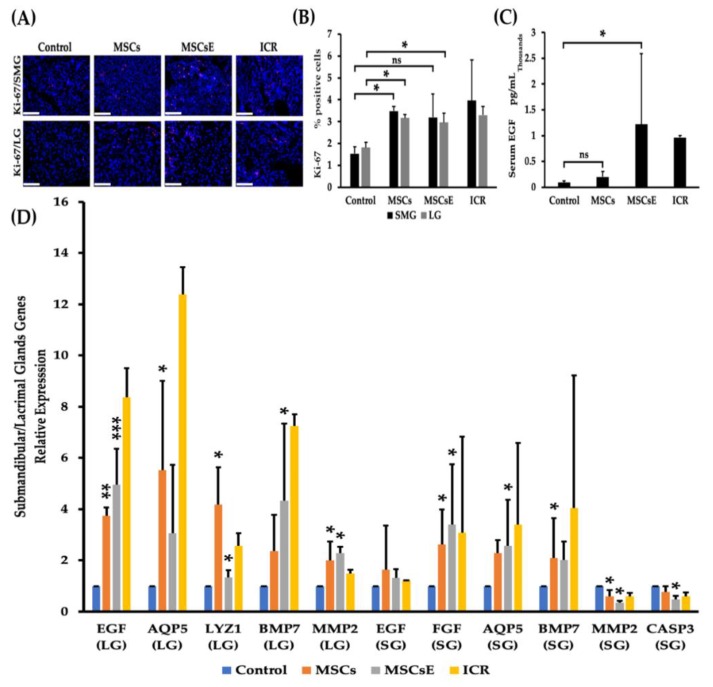
Proliferation rates, serum EGF levels, and gene expression levels of key genes at 16 weeks post-treatment. (**A**) Immunofluorescence staining of submandibular (SMG) (upper panel) and lacrimal (LG) (lower panel) glands for proliferation protein Ki-67 (red), nuclei were stained with DAPI (blue), (**B**) Proliferation rate (Ki-67-positive cells %) for submandibular and lacrimal glands was calculated using random 200× magnified images (acquired by Volocity software) using Image J software. Two examiners independently analyzed images in a blind manner. MSCs/MSCsE treatments promoted tissue proliferation in the glands significantly higher than the control group and their rates were comparable to the ICR group. (**C**) Serum levels of EGF measured by ELISA. Both treatments elevated EGF levels in comparison to the control group, but only MSCsE treatment induced a significantly higher level. (**D**) Relative expression of key genes for tissue function, repair, regeneration, and apoptosis were analyzed by quantitative RT-PCR in lacrimal (LG) and submandibular (SG) glands. Gene expression levels in the lacrimal gland were significantly higher in MSCs-/MSCsE-treated groups than that of the control for AQP5, EGF, LYZ1, MMP2, and BMP7. Gene expression levels in the submandibular gland were significantly higher in MSCs-/MSCsE-treated groups than that of the control for AQP5, EGF, FGF2, and BMP7; and significantly lower for MMP2 and CASP3. Y-axis shows the relative expression of the gene compared to GAPDH, three experimental replicates were conducted for each sample. Scale bar = 74 μm, *p < 0.05, **p < 0.01; ***p < 0.001, *n*= 3–6. All data were presented as mean ± S.D. Control: saline-treated; MSCs: Mesenchymal stem cells; MSCsE: Mesenchymal stem cells extract.

We hypothesized that the trophic and regenerative effects of MSCs/MSCsE treatments are part of the mechanisms that have been implemented. Therefore, proliferation rate, gene analysis, and EGF levels were assessed. Our results showed that the treated groups demonstrated higher proliferation rates and serum EGF (Epidermal Growth Factor) levels, upregulation of several key factors in glandular function/regeneration, and decreased apoptosis. Cell proliferation was evaluated by immunofluorescence staining using the nuclear protein Ki-67 antibody (exclusively expressed in proliferating cells [73]) in salivary and lacrimal glands at 16 weeks post-treatment. Proliferation rates were significantly higher in MSCs-/MSCsE-treated groups (for both glands) compared to control group (Figure 3A,B). Serum EGF levels were evaluated using ELISA. We found that the treatments have successfully contributed and /or induced more EGF secretion in the treated groups, especially MSCsE treatment (Figure 3C). Most tested genes for glandular function/regeneration were also upregulated for both glands in the MSCs-/MSCsE-treated groups when compared to control group (Figure 3D). In the submandibular gland, EGF, FGF2 (Fibroblast Growth Factor 2), AQP5, BMP7 (Bone Morphogenetic Protein 7) genes were all upregulated in the MSCs-/MSCsE-treated groups when compared to the control group. FGF2 gene was ~2.5 and 3 folds higher in MSCs-/MSCsE-treated groups, respectively. The AQP5 gene was upregulated 2.5 folds, which matched its protein expression results obtained by immunofluorescence analysis. However, MMP2 (Matrix Metalloprotienase-2) gene expression gave contradictory results in the MSCs-/MSCsE-treated groups; it was down-regulated in the submandibular glands and upregulated in the lacrimal glands in the MSCs-/MSCsE-treated groups (Figure 3D). CASP3 (Caspase-3) a key gene in the apoptosis process was lower in both treated groups especially in the MSCsE. Gene analysis of lacrimal gland tissue showed significantly higher expression levels for EGF, AQP5, LYZ1 (lysozyme), BMP7, and MMP2 in MSCs-/MSCsE-treated groups compared to the control group (Figure 3D).

### 2.3. MSCs and MSCsE Protected the Cornea Integrity by Preserving its Epithelial Thickness

**Figure 4:**
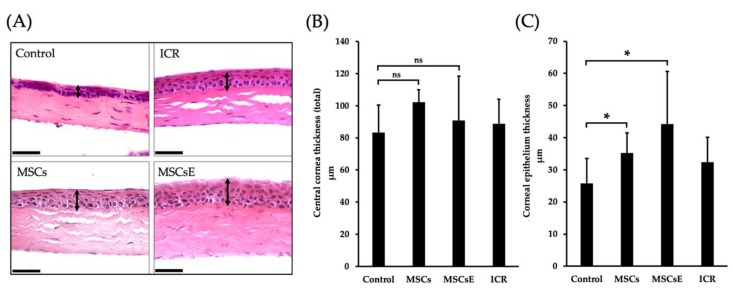
Thickness of the central cornea (total) and the corneal epithelium at 16 weeks post-treatment. (**A**) H&E stained images of the cornea, arrowheads represent the epithelial thickness. (**B**) Analysis of the total cornea thickness. (**C**) Analysis of corneal epithelium thickness. Images of H&E stained sections of the cornea were obtained using Volocity software then Image J was used to assess the thickness. The MSCs-/MSCsE-treated groups showed a significantly higher corneal epithelial thickness. Scale bar = 74 μm, *p ≤ 0.05, *n* = 3–6. All data were presented as mean ± S.D. Control: saline-treated; MSCs: Mesenchymal stem cells; MSCsE: Mesenchymal stem cells extract.

SS patients suffer from corneal thinning as a result of desiccation [74]. Therefore, preservation of the tear secretion will save the cornea from losing its thickness; hence, vision is maintained. We measured the total central corneal thickness and its epithelium alone using serial H&E stained sections (Figure 4A). Total central corneal thickness revealed higher thickness in the treated groups but not statistically significant; however, the epithelial thickness was significantly higher in MSCs-/MSCsE-treated groups when compared to the control (Figure 4).

### 2.4. MSCs/MSCsE Immunomodulatory and Immunosuppressive Functions Were Evidenced by A Decrease in Lymphocytic Influx, A Selective Suppression of B Cells, An Upregulation of IL-10 Secretion and Its mRNA, A Down-Regulation of Gene Expression of Inflammatory Cytokines, and A Lower Levels of Anti-SSA/Ro Autoantibodies

**Figure 5:**
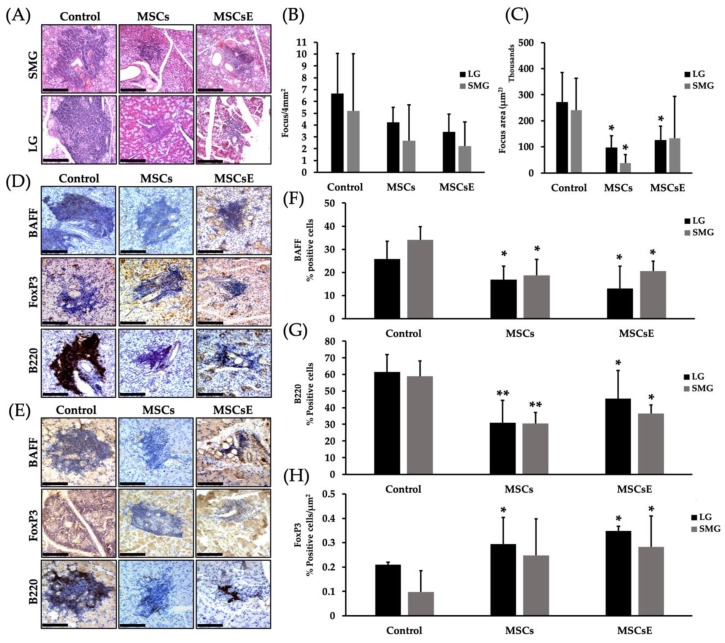
Focus score, focus area and lymphocytes composition analysis in the submandibular (SMG) and lacrimal (LG) glands at 16 weeks post-treatment. (**A**) H&E stained images of lymphocytic infiltrates in the submandibular glands (upper panel) and lacrimal glands (lower panel). (**B**) Focus score analysis (number of lymphocytic infiltrates/4 mm^2^) using serial H&E stained sections, cut at different levels, under the light microscope. The analysis revealed a lower score for the treated groups but was not statistically significant. (**C**) Focus area (in µm^2^) was calculated by Image J software using H&E images (400×/200×) acquired by Volocity software. Treated groups showed significantly smaller focus areas. Immunohistochemical staining of lymphocytic infiltrate for B220 (a pan B cell marker in mice), BAFF (B cells activating factor), and FoxP3 (forkhead box P3, T_reg_ marker) in submandibular glands (**D**) and lacrimal glands (**E**). (**F**,**G**) Quantification of protein expression for BAFF and B220, respectively. The positive signals were measured using Image J software then divided by the size of the lymphocytic infiltrate (focus area). The results were represented as % of signal intensity. (**H**) Quantification of FoxP3^+^ T_reg_ cells. Positive cells were counted in each lymphocytic infiltrate then divided by the focus area (cell/ µm^2^) using Image J software. MSCs/MSCsE groups exhibited a significantly higher FoxP3^+^ and lower B220^+^ and BAFF^+^ cells in the lymphocytic infiltrates when compared to the control group. All images were taken at 200× magnification. Scale bar = 148 μm, *p ≤ 0.05; **p ≤ 0.01, *n* = 3–6. All data were presented as mean ± S.D. Control: saline-treated; MSCs: Mesenchymal stem cells; MSCsE: Mesenchymal stem cells extract.

The efficiency of a therapy against autoimmune diseases relies on its ability to control the immune dysregulation by targeting the pathogenic cells, while leaving the rest of the immune system intact and re-establishing peripheral tolerance [75]. We have assessed the severity of the lymphocytic infiltration by histopathological analysis of serial H&E stained sections of both salivary and lacrimal glands. Results were represented as focus score (number of lymphocytic infiltrate/4 mm^2^, where a focus is an aggregate of ≥ 50 lymphocytes) and focus area (size of the lymphocytic infiltrate (µm^2^)). MSCs-/MSCsE-treated groups showed lower focus score in comparison to the control group; however, due to the small sample size, this difference was not statistically significant.; however, the focus area revealed significantly smaller foci in MSCs-/MSCsE-treated groups (Figure 5A–C). Immunohistochemical analysis of the lymphocytic composition of the glandular infiltrates for B220 (a pan B cell marker in mice), BAFF (B cell Activating Factor) and FoxP3 (Forkhead box P3, a marker for T_reg_) showed significant differences between MSCs-/MSCsE-treated groups and control groups in both lacrimal and submandibular glands (Figure 5D–H). In the submandibular and lacrimal glands: B220^+^ B and BAFF^+^ cells were significantly lower in MSCs-/MSCsE-treated groups compared to the control (Figure 5F,G). FoxP3-rich T_reg_ percentage was significantly higher in MSCs-/MSCsE-treated groups (Figure 5H). 

**Figure 6:**
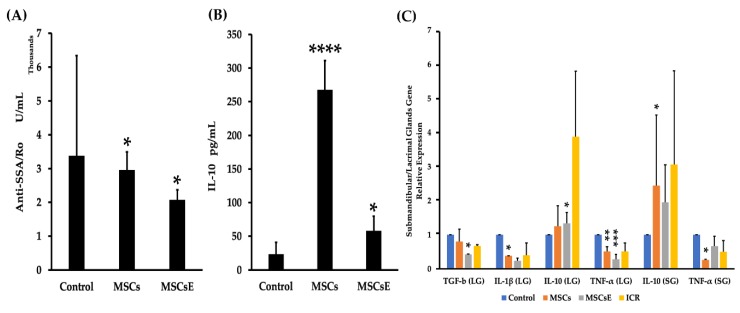
Serum levels of anti-SSA/Ro autoantibodies and IL-10, and gene expression levels of anti-/pro-inflammatory cytokines/factors at 16 weeks post-treatment. (**A**) Serum levels of anti-SSA/Ro autoantibodies (assessed by ELISA) for MSCs-/MSCsE-treated groups exhibited significantly lower levels in comparison to the control group. (**B**) Serum levels of IL-10 (assessed by ELISA) for MSCs-/MSCsE-treated groups were significantly higher than the control group. (**C**) Gene expression levels for anti-/pro-inflammatory cytokines/factors were measured using quantitative RT-PCR in the submandibular and lacrimal glands. MSCs/MSCsE treatments upregulated IL-10 and down-regulated TNF-α gene expressions in both glands, and down-regulated TGF-β, IL-1β in lacrimal glands. Y-axis shows the relative expression of the gene compared to GAPDH, three experimental replicates were conducted for each sample. *p < 0.05; **p < 0.01; ***p < 0.001; ****p ≤ 0.0001, *n*= 4–6. All data were presented as mean ± S.D. Control: saline-treated; MSCs: Mesenchymal stem cells; MSCsE: Mesenchymal stem cells extract.

Serum levels of anti-SSA/Ro and IL-10 were assessed by ELISA. Our results showed lower anti-SSA/Ro (Figure 6A); however, for anti-SSB/La, there was no difference detected between the groups (data not shown). IL-10 levels were expressed significantly higher in the MSCs-/MSCsE-treated groups whilst the control was very low (Figure 6B). Quantitative RT-PCR analysis for anti-/pro-inflammatory cytokines/factors genes in the submandibular and lacrimal glands showed higher gene expression levels for IL-10 and lower levels for TNF-α (Tumor Necrosis Factor alpha) in both glands; whereas, TGF-β (Tumor Growth Factor beta), and IL-1β expression levels were down-regulated in the lacrimal glands of the treated groups in comparison to control (Figure 6C).

## 3. Discussion 

The findings of our study were:

(1) MSCs/MSCsE treatments were successful in preserving the exocrine function of salivary and lacrimal glands in female NOD mice.

(2) Specialized cell subpopulations were preserved in the MSCs-/MSCsE-treated groups along with higher proliferation rates and higher EGF serum levels.

(3) MSCs/MSCsE treatments upregulated expression levels of multiple genes responsible for tissue regeneration, proliferation, and saliva/tears secretion, such as EGF, FGF2, LYZ1 and AQP5 genes and lowered CASP3 a gene involved in the apoptosis cascade.

(4) MSCs/MSCsE treatments promoted the formation of extracellular matrix by upregulation of BMP7 gene expression and prevented fibrosis by down-regulation of TGF-β1 gene expression.

(5) MSCs/MSCsE treatments preserved the corneal integrity by maintaining the epithelial thickness.

(6) Peripheral tolerance in salivary/lacrimal tissues was somewhat restored in MSCs-/MSCsE-treated groups; evidenced by less lymphocytic infiltration (less and smaller foci), selective suppression against B cells, inhibition of anti-SSA/Ro autoantibodies production, and down-regulated levels of pro-inflammatory genes like TNF-α, TGF-β1, and IL-1β.

(7) MSCs/MSCsE treatments influenced immunomodulation via inducing more T-regulatory cells peripherally and upregulation of IL-10.

We have reported in previous studies that BM cells and compact bone-derived MSCs have successfully preserved the salivary gland function when injected into female NOD mice [49,76]. Other researchers have also reported the effectiveness of BM-derived MSCs in treating SS in NOD mice [50]. We have also reported that treatment with bone marrow cell extract (BM Soup) has preserved the salivary gland function, up-regulated the expression of certain critical proteins and genes in female NOD mice [77]. This indicates that the active protein ingredients from BM, including the MSCs subpopulation in it, were preserved and employed successfully when extracted and injected into NOD mice. Hence, combining both principles into MSCsE (mesenchymal stem cells extract) is a unique, safe and a practical treatment modality. The MSCs population in BM is quite small, 0.0017–0.0201% [78]; therefore, their expansion will enable us to enrich our MSCs population pool and accordingly, enrich the extract with more therapeutic proteins [70]. Previous reports have emphasized on the fact that MSCs exert their therapeutic and immunoregulatory capacity via secreting soluble factors in a paracrine mode [79,80]. However, MSCs possess a unique self-renewal and unlimited proliferation capacity that can be unpredictable in vivo [68]. In addition, results from MSCs utilization in clinical trials have been inconsistent and success rates were variable [80]. It was found that the efficacy of these cells is vastly affected by their ability to sense the environment in which they exist [80]. In conclusion, MSCs cell therapy although promising and has been used extensively, but several external and internal factors affect and limit their use. Hence, the development of a safer cell-free biological therapy that compromises the therapeutic capacities of MSCs proteins and avoids their potential risks was our goal. Additionally, the concept of formulating cells into extract is more practical in terms of storage and transfer [81].

The progression of Sjogren’s Syndrome-like disease (SSLD) in NOD mice is divided into three phases. Phase 1 (initiation of glandular pathology) extends from 0–8 weeks of age. It is characterized by cellular disruption in the exocrine glands and initiation of the immune dysregulation. Phase 2 (onset of autoimmunity) extends from 8–16 weeks of age. At this stage, lymphocytic infiltration and autoantibodies formation start. Phase 3 (onset of clinical disease) starts around 16 weeks onwards. In this phase, the secretory loss is very prominent and worsens with time [82]. In our study, we designed the timing and frequency of the treatments to serve several purposes. Regarding the timing, we injected MSCs/MSCsE at 8 weeks of age which is critical in SSLD development in NOD mice. At this age, phase two of SSLD, lymphocytic infiltration and autoantibodies production take place [82]. Therefore, the treatments will combat the immune dysregulation just around the time it starts glandular infiltration and formation of antibodies against its antigens. Moreover, we hypothesized that the MSCsE will reinstate the peripheral tolerance same as the parental cells, MSCs, hoping to ameliorate or at least slow down the disease progression toward the glandular dysfunction (phase three). We tried to choose a time point that is realistic to the pathogenesis timing in SS patients. Yet, it would be more rational to test the treatment at an earlier age in NOD mice, at birth for example (phase one); however, the exact initiation of SS in humans, equivalent to phase 1, is simply unknown to us and it is extremely difficult to investigate. As per the frequency, we aimed at keeping the concentration of MSCs/MSCsE in the blood as high as possible during this phase to allow for a continuous immunomodulation and immunosuppression effects of the treatment while the immune system is actively attacking the glandular tissues. In addition, several studies have reported that MSCs exert a short-lived paracrine effect and this might applies to their extract as well; therefore, repetitive and extended treatment injections is preferred to keep their therapeutic functions for as long as possible [83,84].

MSCs are well documented for promoting tissue repair in addition to their immunosuppression and immunoregulation capacities [60,80,85]. MSCs-/MSCsE-treated groups showed higher SFR/TFR, higher protein intensity for AQP5, AQP4, CK5, α-SMA, and c-Kit, markers for acinar, ductal, myoepithelial, and progenitor/stem cells populations, respectively, in the submandibular and lacrimal glands. Proliferation, detected by Ki-67 antibody, was also upregulated and accompanied by higher serum EGF level, upregulated gene expression of EGF (submandibular and lacrimal glands), FGF2 (submandibular glands), BMP7 (submandibular and lacrimal glands), LYZ1 (lacrimal glands) and MMP2 (lacrimal glands) and down-regulated MMP2 (submandibular glands) and CASP3 (Caspase-3) in the submandibular glands. AQP5 and AQP4 are water channels that are critically involved in the formation of saliva and tears. AQP5 is located at the apical membrane of acinar cells in salivary glands, whereas in the lacrimal glands, it is located apically in the acinar and ductal cells [86,87]. AQP4 is located at the basolateral membrane of acinar cells in salivary glands and laterally in acinar cells of the lacrimal glands [88]. Several studies have reported a defective localization of AQP5 in SS patients and SS mouse models [86]. NOD mice express AQP5 weakly in the lacrimal glands but not the ICR mice, and similar results were found in SS human patients [87,89,90,91]. In the salivary glands, AQP5 tends to be primarily located basolaterally instead of the normal apical location. As assumed, we have found that in the submandibular and lacrimal glands of MSCs-/MSCsE-treated groups the apical expression of AQP5 was upregulated as measured by the immunofluorescence staining whereas in the control group, AQP5 was expressed partially at the apical surface with very low intensity, and the same applies to AQP4. The upregulation of AQPs in the treated mice explains the preservation of the SFR and TFR. Moreover, gene analysis results have confirmed our immunofluorescence staining results. In both glands, AQP5 gene expression was comparable to ICR group, especially in submandibular glands. We have also investigated the expression of several salivary and lacrimal glands markers involved in regeneration and proliferation, including CK5, c-Kit, and Ki-67. CK5 (cytokeratin 5) is an intermediate filament that is widely expressed at birth and considered a marker for ductal/progenitor cells, and CK5^+^ cells are considered a reservoir for the gland regeneration [92]. C-Kit or CD117 (Type III receptor tyrosine kinase) is a marker for stem/progenitor cells in salivary and lacrimal glands [93,94,95]. Ki-67 is a nuclear protein used for detecting actively proliferating cells [96]. MSCs-/MSCsE-treated groups expressed higher CK5^+^, c-Kit^+^, and Ki-67^+^ cells than the control group but slightly less than the ICR group. Lysozyme, secreted by the acinar cells, is a bacteriolytic enzyme responsible for direct defense against bacteria [97,98]. Lysozyme along with lipocalin and lactoferrin compose 80% of the tears proteins [98]. We measured the lysozyme mRNA gene transcripts to evaluate the health and activity of the lacrimal gland. Our results showed an upregulation of lysozyme gene; 4 folds in the MSCs-treated and almost 1.3 in the MSCsE-treated groups, which supports the effectiveness of these treatments in reviving the lacrimal gland. Caspase-3 which is encoded by CASP3 gene, plays an important role in the execution phase of cell apoptosis. Our results showed a down-regulation of CASP3 (submandibular gland) in both treated groups, especially MSCsE, indicating a lower apoptosis. We believe that the general tissue restoration/preservation, the upregulated proliferation, and the downregulated apoptosis are in fact the function of systemic increase of EGF protein and the local upregulation of EGF and FGF2 gene expression levels. 

SS patients suffer from dry eyes (keratoconjunctivitis sicca) due to tear secretion loss from lacrimal glands. The chronic dryness leads to loss of the corneal epithelium, erosions, and a possible perforation if left unmanaged [99,100]. Dry eyes patients displayed thinner central cornea in comparison to healthy subjects [101]. Preservation of the tear secretion is crucial for the health of the ocular apparatus and most importantly, the cornea. Our treatment has successfully preserved the TFR which led to the preservation of the corneal epithelium from the damaging effect of dryness. 

The percentage of B cells in the lymphocytic infiltrates is very crucial for SS patients [102]. Their ratio is higher in advanced cases and in patients with higher focus score. Several studies have reported the ability of MSCs in suppressing B cells, preventing their differentiation, and decreasing their secretion of autoantibodies [67,103]. In our study, we assessed the percentage of B cells in the glandular infiltration by measuring the intensities of B220^+^ (a pan B cell marker in mice) B cells and the intensity of BAFF using immunohistochemical staining. We also measured the serum levels of the autoantibodies anti-SSA by ELISA. BAFF is important for maturation and homeostasis of B cells; however, uncontrolled secretion leads to autoimmunity [104]. Excess BAFF will enable autoreactive B cells to overcome apoptotic signal in negative selection. It is expressed at high levels in several autoimmune diseases, including SS. A positive correlation was found between the levels of BAFF and autoantibodies especially anti-SSA in SS patients [105]. Our analysis showed a significantly lower intensities for B220^+^ B cells and BAFF in the submandibular and lacrimal glands foci of the MSCs-/MSCsE-treated groups. BAFF is produced by several immune cells, including T lymphocytes and dendritic cells [106]. We believe that the immunosuppressive action of MSCs-/MSCsE on these cells has resulted in less production of several factors, including BAFF. The reduction of BAFF expression and/or blocking its action have led to the reduction of B cells survival signals. The later eventually steered the reduction in autoantibodies secretion by plasma cells [107]. In conclusion, because we achieved comparable results from both treatments, we believe that the extract contains enough factors and cytokines that are necessary and efficient for the immunomodulation needed in SS. 

TNF-α and IL-1β genes were upregulated in SS patients and NOD mice [108,109]. Activated macrophages secretion of TNF-α and IL-1β was attenuated by MSCs [110]. We have previously reported that MSCs down-regulated TNF-α gene expression in treated NOD mice [49]. Our results showed a down-regulation of gene expression for the pro-inflammatory cytokines: TNF-α, and IL-1β in the MSCs-/MSCsE-treated groups in comparison to the control group. However, MSCsE-treated group showed lower expression than MSCs treated group. We believe that the protein composition in the extract was more efficient in the suppression mechanism, probably, due to the direct bioavailability of the proteins in more significant quantities than what the MSCs can secret to achieve the same results. 

Several studies, including ours, have reported that MSCs-based treatment has increased the percentage of FoxP3^+^-T_reg_ [49,85]. FoxP3^+^ T_reg_ cells are essential in self-tolerance, tissue repair and proliferation [111,112]. T_reg_ enforces immune suppression either by direct effect on antigen presenting cells like dendritic cells, or via anti-inflammatory cytokines secretion, such as IL-10 and TGF-β1 [113,114]. IL-10 is a potent immunosuppressive cytokine that can act through different channels to block immune dysregulation, such as inhibition of pro-inflammatory cytokines TNF-α and IL-1β, antigen presentation and immune cells proliferation [115,116]. IL-10 is secreted by various T cell populations most importantly FoxP3^+^-T_reg_ and it is also secreted by MSCs to target specific cells, including T_reg_ [117]. Our results showed a significant increase in the percentage of FoxP3^+^-T_reg_ within the lymphocytic infiltration in the submandibular and lacrimal glands of MSCs-/MSCsE-treated groups in comparison to the control group. We have also found an upregulation of IL-10 serum levels and its mRNA, down-regulation of TNF-α mRNA and IL-1β in submandibular and lacrimal glands. Aggarwal et al. 2005 have reported that hMSCs have induced a more anti-inflammatory and tolerogenic environment [118]. They found that hMSCs have negatively influenced DC1 secretion of TNF-α, prompted DC2 to secrete more IL-10, and caused an increased in T_reg_ cell number. Thus, the high increase in IL-10 and its mRNAs was orchestrated by MSCs. MSCs-secreted IL-10 has positively influenced T_reg_ which in its turn secreted more that has led to immune tolerance induction via a cascade of regulatory steps. Although IL-10 serum levels in MSCsE-treated group was significantly higher than that of the control but it was lower than the MSCs-treated group levels. We think that this difference is due to the constant release of Il-10 from the MSCs via a paracrine mode from their engraftment site. However, our quantitative RT-PCR results showed lower levels of TGF-β1 in MSCs and even much lower in MSCsE-treated group in comparison to the control group. TGF-β play an important role in the salivary glands morphogenesis, extracellular matrix deposition, and controls the immune homeostasis as well [119,120]. Mice that were overexpressing TGF-β suffered hyposalivation due to the excessive deposition of fibrous tissue in the gland [119]. The therapeutic level achieved by our treatments has played its role in the anti-inflammatory aspect; orchestrated by inducing IL-10 from DC2 and T_reg_ and prevented the over formation of extracellular matrix and eventual fibrosis that might have occurred if excessive TGF-β1 was secreted. MSCs/MSCsE have delivered a balanced treatment that managed the immune dysregulation and promoted tissue restoration and regeneration. In agreement with our finding, Park et al. have reported that conditioned media from human umbilical cord blood-MSC down-regulated TGF-β1 and upregulated BMP7 levels in renal epithelial cells [121].

## 4. Materials and Methods

### 4.1. Animal Models

All experimental procedures were performed following the guidelines imposed by the Canadian Council on Animal Care. Our protocol (2007-5330) was approved by the University Animal Care Committee (UACC) at the McGill University.

#### 4.1.1. Recipient

Eight-week-old female NOD mice with Sjogren’s-like disease purchased from Taconic Farms (Germantown, NY, USA) were randomized into three groups. Group 1: NOD mice treated with bone marrow-derived MSCs (*n* = 8), Group 2: NOD mice treated with bone marrow-derived MSCsE (*n* = 12) and Group 3, Control: NOD mice treated with normal saline (*n* = 11).

#### 4.1.2. Donors

Eight-week-old male C57BL/6 were purchased from Jackson Laboratory (Bar Harbor, ME, USA). Mice were kept at the animal facility according to the animal care rules and regulation.

#### 4.1.3. Wild Type Control

Eight-week-old ICR (Institute of Cancer Research) mice were purchased from Taconic Farms (Germantown, NY). ICR is an outbred strain from which NOD subline was derived [50,122]. This strain was used as a wild type and SS-free control mouse model. ICR mice received no treatment. Their salivary and tear flow rates were measured at the same time the treated and control NOD groups were (*n* = 6).

### 4.2. Blood Glucose Monitoring

Starting at 12 weeks of age, fasting blood glucose was monitored for all mice once weekly using Accu-Check^®^ (Roche) system where mice were bled at the tail middle third. All mice with diabetes (> 250 mg/dL) were administered insulin subcutaneously and checked daily afterwards. All mice with borderline (150–250 mg/dL) level were checked twice weekly. Mice with normal glucose levels were monitored weekly until hyperglycemia was evidenced then the above protocol was followed.

### 4.3. Mesenchymal Stem Cells (MSCs) Culture, Preparation of the Extract (MSCsE), and Their Transplantation

Bone marrow (BM) cells were harvested from 8-week-old male C57BL/6 mice. Briefly, mice were euthanized according to the animal ethics protocol at McGill University. Mice were disinfected thoroughly with 70% ethanol then the femur and the tibia of the hind limbs were obtained [52]. The isolated bones were washed with cold 1× PBS to remove any remaining blood or tissues and kept on ice throughout the procedure. The bones were placed inside a pre-cut sterilized 1.0 mL pipette tip which was adapted inside a sterilized 1.5-mL Eppendorf tube. The Eppendorf tubes were then placed in a 4 °C centrifuge at a speed of 4040× *g* for 30 s then the pellets were collected and kept on ice. The BM pellet was reconstituted and mixed thoroughly with the MSCs media (MesenCult™ MSC Basal Medium+ MesenCult™ Mesenchymal Stem Cell Stimulatory Supplements (STEMCELL)). The cell suspension was filtered using a 40-µm nylon cell strainer. The cell number was determined then the cells were cultured in 75 cm^2^ flasks (Corning Inc, Corning, NY, USA) and seeded at a ratio of 40 × 10^5^ cells/cm^2^. The flasks were closed tightly and incubated unmoved for five days at 37 °C in a 5% CO_2_ incubator. Thereafter, floating cells were discarded, and a fresh media was added. When the cells (spindle in shape) reached 70–80% confluency, they were passaged at a ratio of 1:3. MSCs were enriched by passaging as the non-MSCs and hematopoietic cells tend to attach strongly to the culture vessels. When cells reached passage 8, they were detached and prepared for treatment. Each mouse received 2.0 × 10^6^ cells/100 μL in normal saline once weekly for four consecutive weeks starting at 8 weeks of age via the tail vein.

For MSCsE preparation: cells from passage 8 were reconstituted in normal saline at a ratio of 2.0 × 10^6^ cells/100 µL. The cells suspension was placed in liquid nitrogen to freeze then thawed at room temperature; the process was repeated three times to ensure complete cell rupture. At the end of the third cycle, the tubes were thawed and placed in the centrifuge at 4545× *g* speed for 30 min at 4 °C temperature. At the end of the centrifugation cycle, the supernatant (i.e., the cell extract) was collected for immediate use or stored at −80 °C freezer. Treated mice received 100 µL of MSCsE once weekly for four consecutive weeks starting at 8 weeks of age. 

### 4.4. Secretory Function of the Salivary and Lacrimal Glands (Salivary Flow Rate: SFR and Tear Flow Rate: TFR)

Secretory function of the salivary glands (Salivary Flow Rate: SFR) was measured by inducing mild gas anesthesia in NOD mice using 1.5–3% isoflurane, 5% halothane and 1 L/min oxygen. When the mice were sedated, SFR was stimulated by injecting 1.0 mg pilocarpine/kg body weight subcutaneously in the dorsal side of the neck. Whole saliva was obtained from the oral cavity by placing a micropipette into a pre-weighed 0.5 mL microcentrifuge tubes at the corner of the mouth. Five minutes after the pilocarpine injection, saliva collection was done for 10 min; however, any saliva produced in the first 5 min was discarded. Saliva volume was determined gravimetrically and then stored in -20 °C freezer. SFR was measured pre-treatment at 8 weeks of age (week 0) then at 4, 8, 12, and 16 weeks post-treatment.

TFR was measured at the same appointment as SFR to reduce the animal discomfort. After 10 min of the injection of pilocarpine, red phenol threads (Zone Quick, FCI Ophthalmics, Japan) were placed gently in the medial canthus of both eyes with the aid of fine tip tweezers for 5 min; any tear secretion in the previous 10 min was removed before the final measurement was recorded. The thread was measured by a ruler to the approximate mm and then placed in a sterilized 1X PBS containing tube and stored in −20 °C freezer. The readings of both eyes were averaged, and then a mouse group average was calculated. Only mice that showed lymphocytic infiltration in the lacrimal gland were included in the TFR, lacrimal focus score, lacrimal focus area, and corneal thickness assessments in the results. The percentage of positive mice for lacrimal gland infiltration were 40–75% among the groups.

### 4.5. Submandibular and Lacrimal Gland Tissue and Serum Analysis

Al analysis were carried out 16 weeks post-treatment (24-week-old NOD mice).

#### 4.5.1. Serum Preparation and Analysis

Shortly after the animals were euthanized, the blood was drawn via cardiac puncture. Blood was left to clot for 30 min at room temperature then centrifuged at 1212× *g* for 8 min. Serum was isolated, aliquoted and then stored in −80 °C freezer for further analysis later. ELISA was used for the analysis of serum EGF (ab100679, abcam), Anti-SSA/Ro (5710, Alpha Diagnostics), anti-SSB/La (5810, Alpha Diagnostics), and IL-10 (ab46103, abcam).

#### 4.5.2. Focus Score and Focus Area

Focus score is defined as the number of the lymphocytic infiltrates/4mm^2^, where a focus is an aggregate of ≥ 50 lymphocytes). It is evaluated under the microscope using serial H&E stained histological sections cut at different levels. Focus area defined as the area occupied by the lymphocytic infiltrates in the glands measured in µm^2^. It was performed by using 200 or 400× magnified images that were acquired using Volocity software. Thereafter, the size of each focus was assessed using ImageJ software. The average for each group was then calculated and represented in µm^2^.

#### 4.5.3. Immunohistochemistry

Formaldehyde-fixed paraffin-embedded (FFPE) submandibular and lacrimal glands sections were blocked for endogenous peroxidases by using fresh 3% H_2_O_2_ after the antigen retrieval step with acetic acid pH 6. The nonspecific binding of the primary antibodies was blocked with 1% BSA and 5% normal goat serum in PBS for one hour at room temperature. The primary antibodies, B220 (550286, BD Biosciences), FoxP3 (14–5773, eBioscience), BAFF (11021244, Enzo Life Sciences) were incubated overnight in 4 °C refrigerator. Polyclonal rabbit anti-Rat secondary antibody was applied for 1 h at room temperature. Visualization was performed using the DAB+ system (k3468, Dako). Counter-staining with hematoxylin stain was run for one minute. Brightfield microscopy was used to obtain magnified images using Volocity software. The percentage of positive cells was performed using Image J software. For B220 and BAFF analysis, the positive signal was measured as the intensity and was divided by the focus area (surface area of the lymphocytic infiltrate) and an intensity percentage was generated. For FoxP3, the positive cells were counted then divided by the total area of the focus then the data was represented as cell/µm^2^. Data were represented as mean ± S.D.

#### 4.5.4. Immunofluorescence

Submandibular and lacrimal glands frozen sections were blocked with 1% BSA and 5% normal donkey serum in PBS for one hour at room temperature. The primary antibodies: AQP5 (ab78486, abcam), AQP4 (ab9512, abcam) CK5 (PRB-160P, Covance), α-SMA (ab7817, abcam), c-Kit (ab5506, abcam), Ki-67 (9129S, Cell Signaling Technology) were incubated for 24 h in 4 °C refrigerator. Polyclonal donkey anti-mouse or rabbit fluorophore-conjugated secondary antibodies in 1X PBS were applied for 1 h at room temperature. Finally, 4′,6-diamidino-2-phenylindole dihydrochloride (DAPI) (d1306, Invitrogen) was applied for 3 min. Images were acquired using Volocity software and the intensity was analyzed using 4–6 200× magnified fields using ImageJ. Using ImageJ software, the positive signal occupying area was calculated then divided by the total area of the tissue and a percentage was generated. An average per mouse then per group were calculated and represented as mean ± S.D.

#### 4.5.5. Quantitative Real-Time PCR

Total RNA extraction was performed with PureLink RNA Mini kit (Thermofisher:12183018A). High-Capacity cDNA Reverse Transcription kit (Thermofisher:4368814) was utilized to create the cDNA strands. Triplicate quantitative RT-PCR assays were performed by Step One Plus (Life Technologies) in TaqMan Universal Master Mix II (4440040, Applied Biosystem, Foster City, Canada). The probes used were: EGF (assay ID: Mm00438696), AQP5 (assay ID: Mm00437578), BMP7 (assay ID: Mm00432102), FGF2 (assay ID: Mm00433287), IL-10 (assay ID: Mm01288386), IL-1β (assay ID: Mm00434228), TNF-α (assay ID: Mm00443258), TGF-β1 (assay ID: Mm01268596), MMP2 (assay ID: Mm00439498), LYZ1 (assay ID: Mm01228256), Caspase-3 (assay ID: Mm01195085) and GAPDH (Glyceraldehyde-3-phosphate dehydrogenase, assay ID: Mm99999915) was used as an endogenous reference gene. Three experimental replicates were performed for each sample. PCR was run at 50 °C for 2 min, 95 °C for 10 min, and 40 cycles (95 °C for 15 s, 60 °C for 1 min).

#### 4.5.6. Central Cornea Thickness Analysis

Both eyeballs were removed at the time of euthanasia and prepared for FFPE procedure. The eyeball blocks were cut until reaching the center; afterwards, serial 7 µm thickness sections were obtained and stained with H&E. Using Volocity software, 200× images were acquired for the cornea. With the aid of ImageJ software, the total thickness of the cornea (epithelium+ connective tissue) and the epithelium alone were measured centrally. Group average was calculated and represented in µm.

#### 4.5.7. Statistical Analysis

To determine statistical significance, we used one-way ANOVA test (*p* < 0.05) by GraphPad Prims version 7 was performed for control-, MSCs- and MSCsE-treated groups.

## 5. Conclusions

This study reveals the therapeutic benefits of MSCsE in treating SSLD in NOD female mice. Our study show very promising results that are comparable to MSCs treatment. The safety, bioavailability, convenience of use, and transference are all advantages that MSCsE can offer in comparison to MSCs. However, further investigations are required to assess further possible mechanism of action. In addition, the extract comprises many constituents, most of which are proteins; therefore, more exploration on its composition will be beneficial.
