# Correction: Abughanam, G., et al. Mesenchymal Stem Cells Extract (MSCsE)-Based Therapy Alleviates Xerostomia and Keratoconjunctivitis Sicca in Sjogren’s Syndrome-Like Disease. *Int. J. Mol. Sci.* 2019, *20*, 4750

**DOI:** 10.3390/ijms22020894

**Published:** 2021-01-18

**Authors:** Ghada Abughanam, Osama A. Elkashty, Younan Liu, Mohammed O. Bakkar, Simon D. Tran

**Affiliations:** McGill Craniofacial Tissue Engineering and Stem Cells Laboratory, Faculty of Dentistry, McGill University, Montreal, QC H3A 0C7, Canada; ghada.abuelghanam@mail.mcgill.ca (G.A.); osama.elkashty@mail.mcgill.ca (O.A.E.); Younan.liu@mcgill.ca (Y.L.); mob11@case.edu (M.O.B.)

The authors wish to make the following corrections to this paper [1]:

Figure 2B was mistakenly replaced by Figure 2D, the corrected Figure 2 is shown below:


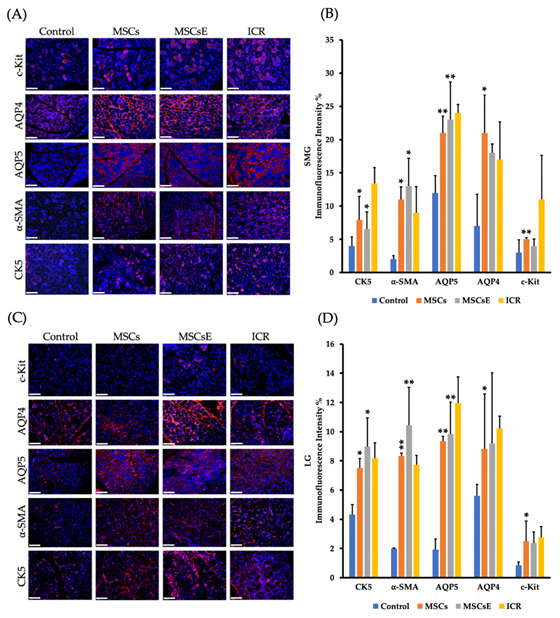


We apologize for any inconvenience brought to the readers.
